# Single-shot technique of cryoablation for atrial fibrillation has comparable effective and safety outcomes compared to standard technique: insights from multiple clinical studies

**DOI:** 10.3389/fcvm.2023.1195492

**Published:** 2023-09-07

**Authors:** Changjian He, Wenchang Zhang, Lei Yin, Mingzhuang Sun, Zihan Zhao, Guojie Ye, Tengfei Liu, Wence Shi, Da Zhang, Feng Li, Chunhua Ding

**Affiliations:** ^1^ Cardiac Department, Aerospace Center Hospital (Peking University Aerospace School of Clinical Medicine), Beijing, China; ^2^ Department of Cardiology, The Second Hospital of Hebei Medical University, Shijiazhuang, China; ^3^ Department of Cardiology, The Second Affiliated Hospital of Soochow University, Suzhou, China

**Keywords:** atrial fibrillation, cryo-balloon ablation, single-shot technique, meta-analysis, time to isolation

## Abstract

**Background:**

Although there are many freezing protocols available, the optimal freezing dose is still not determined. We aimed to evaluate the effectiveness and safety of different freeze strategies of CBA in the treatment of AF.

**Methods:**

PubMed, Cochrane Library, Web of Science, and Embase were searched up to 1st December 2022. Studies comparing the outcomes between single-shot technique and standard technique of cryoablation were included. Subgroup analysis identified potential determinants for single-shot technique procedure.

**Results:**

Our search resulted in 3407 records after deduplication. A total of 17 qualified studies met our inclusion criteria. Compared with standard technique, single-shot technique of cryoablation has a comparable rate of freedom from AF/AT(RR 1.00; *P *= 0.968), a trend for lower rate of procedure complications (RR 0.80; *P *= 0.069), a lower rate in transient phrenic paralysis (t-PNP) (RR 0.67; *P *= 0.038), a similar rate in persistent phrenic paralysis (per-PNP) (RR 1.15; *P *= 0.645), as well as a comparable procedure parameters. Importantly, potentially significant treatment covariable interactions in procedure complications were found in freeze strategy subgroup, male proportion subgroup and age subgroup, including single-shot freeze (RR 1.02; *P *= 0.915) and TTI-guided (RR 0.63; *P *= 0.007) with interaction *P *= 0.051, high male proportion (RR 0.54; *P *= 0.005) and a low male proportion (RR 0.94; *P *= 0.759) with interaction *P *= 0.074, as well as age ≥ 65 (RR0.91; *P *= 0.642) and age <65 (RR 0.54; *P *= 0.006),interaction *P *= 0.090. Meanwhile, only one significant treatment covariable interactions in procedure complications was found in the hypertension subgroup, including HT > 60% (RR 0.89; *P *= 0.549) and HT ≤ 60% (RR 0. 46; *P *< 0.01) with interaction *P *= 0.043.

**Conclusions:**

Our study suggested that single-shot technique of cryoablation has comparable effective and safety outcomes for AF ablation compared to standard technique.

## 1. Introduction

Pulmonary vein isolation (PVI) is the cornerstone of catheter ablation in patients with atrial fibrillation (AF). Cryo-balloon ablation (CBA) has been widely used for AF treatment worldwide. Whether in patients with paroxysmal atrial fibrillation (PFA) or persistent atrial fibrillation (per-AF), PVI alone has achieved satisfactory curative effects (1–3), which is superior to drug therapy and not inferior to radiofrequency ablation(RFA). CBA is characterized by less procedural complexity and a shorter learning curve. Different center and operator experience had a weaker impact on program success in CBA than in RFA procedure (4). Recently, several studies have demonstrated high acute efficacy and low complication rates using CBA for PVI in paroxysmal or persistent AF in both low-volume and high-volume centers (5, 6).

Due to the cumbersome nature of RFA to achieve PVI, CBA was developed to further simplify the PVI procedure by homogenizing the results of operators with different experience. However, the ideal energy dose has not yet been standardized, and preclinical and clinical studies have been trying to find the optimal cryo-energy dose that results in durable PVI with the shortest effective cryo-duration. The current CBA protocol are varied, including fixed cycle single ablation or double ablation, cryo-dose guided by TTI, and cryo-dose based on temperature monitoring (7, 8). This may hinder its application and promotion, and often confuses beginners, thus affecting the safety and effectiveness of CBA. The latest CB−2 ablation studies have demonstrated high single-procedure success rates for PVI, with good long-term clinical outcomes even when “no addition” cryo-protocols or shorter cryo-cycles were applied (9–15). Moreover, the rate of persistent PVI was high in patients who underwent re-acceptance of atrial tachyarrhythmias ablation after CB-2 ablation (16, 17). In addition, recent studies have shown that time-to-isolation (TTI) has the potential to be the best indicator for cryo-dose monitoring and a strong marker for predicting acute and durable PVI (18, 19). However, the current ability to monitor TTI needs to be further improved, and the TTI protocol has not yet formed a standardized unified scheme.

Therefore, in this study, we aimed to evaluate the effectiveness and safety of different administration strategies of CBA in the treatment of AF.

## 2. Methods

### 2.1. Search strategy

We conducted this systematic review according to the Preferred Reporting Items for Reviews and Preferred Reporting Items for Systematic reviews and Meta-Analyses (PRISMA) guidelines (20). The study protocol was registered in the PROSPERO database (Registration ID: CRD42022382336).

A comprehensive literature search was conducted with four online search engines, including PubMed, Cochrane Library, Web of Science, and Embase, by two independent reviewers (C.J.H and F.L) from the establishment of the databases up to 1st December 2022. Search keywords included “cryoballon”, “cryoablation”, “ablation”, and “atrial fibrillation”. Trials comparing the therapeutic effects between single-shot technique of cryoablation and standard technique in patients with AF were included. In addition, the reference list of review literature and retrieved eligible literature were hand-searched for potential publications not being identified previously.

### 2.2. Study design

A clinical study was eligible if it met the following inclusion criteria: (1) randomized controlled trials and cohorts, observational studies, and case controls. (2) studies comparing the results of different freezing strategies, including long-term freedom of atrial fibrillation / AT, phrenic paralysis, pericardial effusion, stroke / transient ischemic attack (TIA) and surgery-related death. Phrenic nerve palsy (PNP)was classified as either transient or persistent. Transient phrenic nerve palsies (t- PNP), defined as palsy resolution by the end of the cryo-balloon procedure or before discharge, whereas persistent phrenic nerve palsy (per-PNP) was characterized by continued loss of phrenic nerve function that persisted during follow-up. (3) full-text studies published in English in peer-reviewed journals. (4) for multiple publications of the same trial or cohort, only the research containing the most data is included. Meanwhile, single-arm study, studies without original data, review articles, case reports, letters, editorials, and animal studies were excluded. Two independent reviewers (C.J.H and F.L) searched and reviewed the titles, abstracts, and full texts to determine the eligible study. Any disagreements about eligibility were resolved by consulting a third reviewer (C.H.D).

The intervention evaluated was CBA for AF. In this study, the single-shot technique was defined as the AF patients underwent single-shot freeze or TTI-guided single freeze. The standard technique was defined as the AF patients underwent double freeze or bonus freeze. Moreover, single-shot technique was allowed for additional freeze to reduce the concerns about lesion durability when sensory isolation was not achieved or achieved lately in the freezing process.

### 2.3. Data extraction

For each eligible study, data were extracted by two independent researchers (C.J.H and F.L), and any differences were resolved through discussion with a third researcher (C.H.D). We first recorded the eligible characteristics of the study, including the first author, year of publication, study design, number of patients, and follow-up time. At the same time, the demographic and clinical characteristics of the patients and the indicators related to the procedure were also recorded.

### 2.4. Quality assessment

Given the heterogeneity of the eligible studies, the quality of each study was assessed using two different critical appraisal tools by two independent researchers. For randomized clinical trial included in our review, the Cochrane risk of bias assessment tool was used. The Newcastle-Ottawa Quality Assessment Scale (NOS) was used to assess observational studies. We also assessed the potential publication using Egger's and Begg's test.

### 2.5. Statistical analysis

Categorical variables were presented as frequencies or percentages, and continuous variables were presented as means ± standard deviations, or median with interquartile range, as appropriate. The Stata version 16.0 (College Station, Texas 77845 USA, StataCorp LP) was used for all statistical analyses, and *P* < 0.05 was considered statistically significant.

We used *I*^2^ to quantify the proportion of variance derived from between-study heterogeneity (21) and *I*^2^ values of 0, <25%, 25%–49%, and >50% were considered as no, low, moderate, and high heterogeneity, respectively. If *I*^2^ value was more than 50%, a random effect model was adopted. Otherwise, a fixed effect model was used. Meanwhile, when significant heterogeneity was presented, we performed a sensitivity analysis to examine the effect of a single study on the overall risk estimate by sequentially omitting one study at a time. We also assessed the potential publication bias using Egger's tests.

In addition, subgroup analysis was performed to screen sources of heterogeneity and potential determinants of AF ablation outcomes between single-shot technique of cryoablation and standard technique. According to the characteristics of eligible studies, some potential factors, and previously reported factors, a total of nine subgroup factors were identified, including study design, follow-up time, sample size, age cutoff, male proportion, hypertension (HT) proportion, paroxysmal atrial fibrillation (PAF) proportion, left atrial diameter (LAD) and freeze strategy. If the study design included only one center, it was defined as a single-center subgroup; otherwise, it was defined as a multicenter subgroup. Follow-up time was divided into two subgroups (>12 months and ≤12 months). According to the cutoff value 100 and 65, the group sample size and the age cutoff were divided into two subgroups, respectively. According to cutoff values of 60%, the male proportion and HT proportion were divided into two subgroups, respectively. According to the cutoff value 100% and 40, the PAF proportion and LAD were divided into two subgroups, respectively. Based on the freeze strategy, single-shot and TTI-guided subgroups were defined.

## 3. Results

### 3.1. Study selection and quality assessment

**Figure 1:**
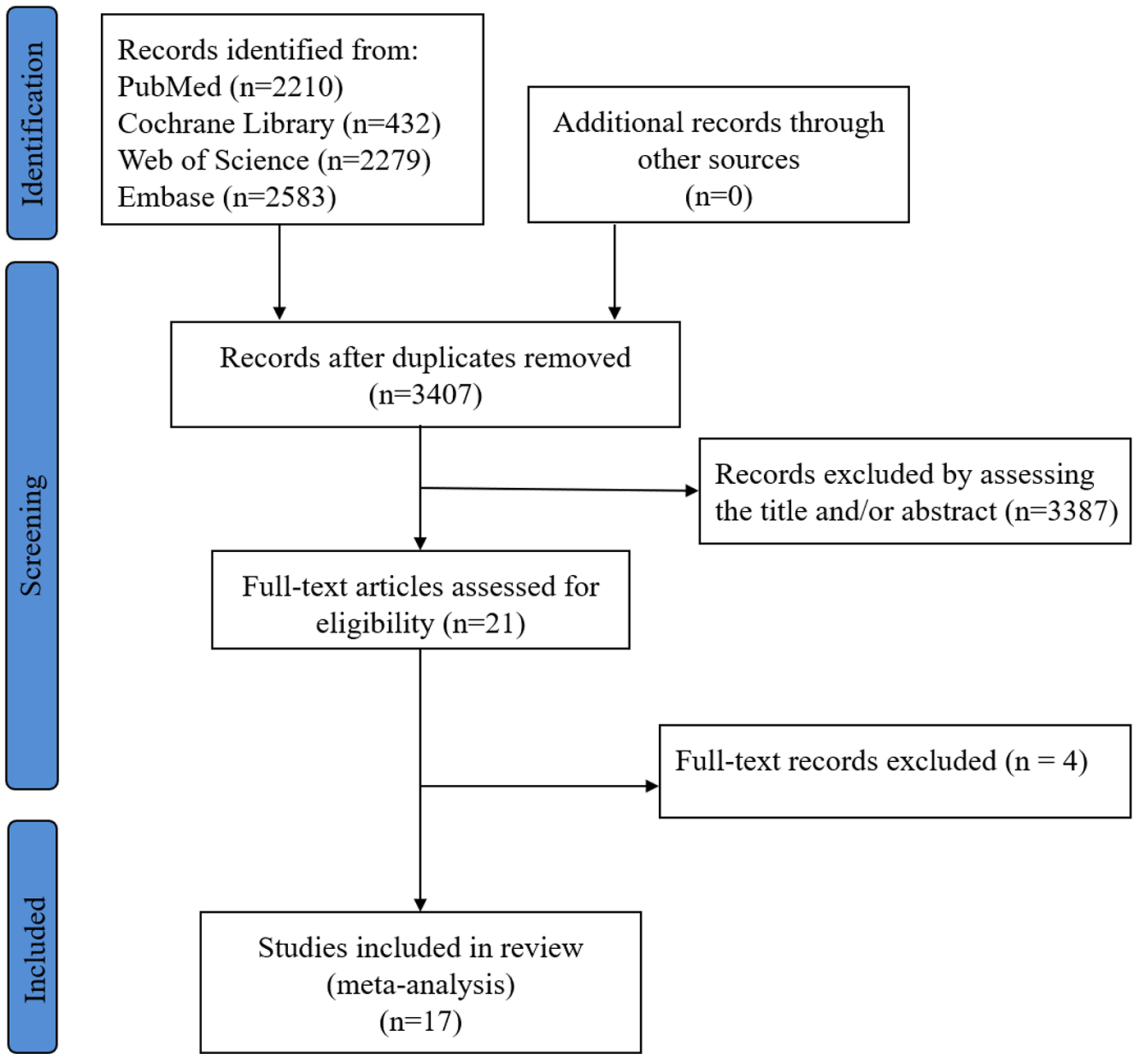
Flow diagram of the study selection.

The research selection flow chart is shown in Figure 1. Our search resulted in 3,407 records after deduplication. Abstracts were independently screened by two investigators. Finally, a total of 17 qualified studies met our inclusion criteria, including 6 prospective studies (15, 22–26) and 11 retrospective studies (8, 13, 14, 17, 27–33), including 4,688 patients with AF (2,526 in single-shot technique group and 2,162 in standard technique group). In two multicenter studies (17, 29), the only two subgroups, including bonus-freeze protocol group and the time-to-effect protocol group, were extracted due to their comparability. The demographic and clinical characteristics was presented in Table 1. Details of cryoablation are shown in Table 2.

**Table 1:** The demographic and clinical characteristics.

First author, year, reference	Study design	Center	Sample size		Gender (male %)		Age		Hypertension (%)		Paroxysmal AF (%)		LAD (mm)	
			Single-shot technique	Standard technique	Single-shot technique	Standard technique	Single-shot technique	Standard technique	Single-shot technique	Standard technique	Single-shot technique	Standard technique	Single-shot technique	Standard technique
Ferrero-2021	Prospective study	Multicenter center	679	553	NA	NA	NA	NA	NA	NA	NA	NA	NA	NA
Bianchini- 2021	Retrospective study	Single center	80	80	58.8	57.5	66.9 ± 11.8	67.6 ± 12.5	72.5	71.3	66.2	78.7	40.8 ± 5.3	40.8 ± 6.2
Heeger-2- 2020	Retrospective study	Multicenter center	94	31	39.4	49.4	66	64	67.0	55.0	45.7	77.4	45.0	44.0
Cordes-2019	Prospective study	Single center	35	35	71.4	80.0	58	60	62.9	42.9	60	57.1	NA	NA
Miyamoto-2019	Prospective study	Multicenter center study	55	55	65.5	63.6	63.1 ± 11.8	64.0 ± 11.0	41.8	47.3	100	100	37 ± 7	38 ± 6
Koektuerk-2019	Retrospective study	Single center study	77	92	63.6	85.9	61 ± 10	64 ± 10	49.4	57.6	100	100	32.5 ± 8.5	31.1 ± 9.4
Vallès-2018	Retrospective study	Single center study	88	69	64.8	76.8	55.7 ± 8.7	53.8 ± 10.2	37.9	40.3	79.5	69.6	41.6 ± 6.6	41.7 ± 5.7
Yoshiga-2019	Retrospective study	Single center study	67	33	58	69.7	65.1 ± 10.0	67.5 ± 8.3	52.2	51.5	100	100	39.0 ± 5.6	38.7 ± 5.5
Pott-2018	Retrospective study	Single center study	100	100	56.0	57.0	65.0 ± 10.9	65.3 ± 11.3	72.0	76.0	65.0	69.0	45 ± 7	43 ± 6
Rottner-2018	Retrospective study	Multicenter center study	352	59	NA	NA	NA	NA	NA	NA	NA	NA	NA	NA
Ströker-2018	Retrospective study	Multicenter center study	256	256	62	67	59 ± 12	60 ± 11	NA	NA	82	79	43 ± 10	42 ± 6
Mortsell-2018	Prospective study	Single center study	69	70	69.6	77.1	61.9 ± 9.08	68.3 ± 10.0	42.0	50.0	49.3	40	NA	NA
Aryana-2017	Prospective study	Multicenter center study	355	400	69	74	64 ± 11	63 ± 11	58	58	72	74	43 ± 7	48 ± 6
Chun-2016	Prospective study	Single center study	50	50	60	58	66 ± 10	63 ± 12	70	72	100	100	41 ± 4	39 ± 4
Ekizler-2017	Retrospective study	Single center study	56	80	57	55	58 (48–67)	62 (49–68)	45	44	100	100	38 (36–42)	39 (36–42)
Tebbenjohanns-2016	Retrospective study	Single center study	53	139	51	54	66 + 10	61 + 11	NA	NA	72	63	40 + 6	41 + 7
Heeger-1	Retrospective study	Single center study	60	60	63	60	61 ± 11	62 ± 11	62	70	83	75	4 2 ± 8	43 ± 5

**Table 2:** Details of CBA.

First author, year, reference	Freeze-protocol		Total procedure time (min)		Freeze-time (min)		Follow-up (months)
	Single-shot technique	standard technique	Single-shot technique	standard technique	Single-shot technique	standard technique	
Ferrero et al.-2021	cryoapplications duration was 180 or 240 s	an extra application of cryothermia was carried out after the application that had blocked the pulmonary vein.	111.8 ± 40.55	115.6 ± 42.12	19.6 ± 7.21	24.4 ± 8.04	12
Bianchini et al.- 2021	TTI < 75”,Single 240 s application, no bonus freeze; TTI >75”,120 s bonus freeze	TTI < 75”,Double 240 s application, no bonus freeze; TTI > 75”,180 s bonus freeze	56.7 ± 15.4	59.7 ± 16.7	NA	NA	Not mentioned
Heeger-2 et al.- 2020	TTI + 120 s, If no real-time PV signal recording could be obtained, an empiric freeze cycle duration of 180 s was applied.	comprised a fixed freeze-cycle duration of 240 s, followed by an additional bonus-freeze of 240 s after successful PVI	86.67 ± 28.4	123.4 ± 31.5	NA	NA	Not mentioned
Cordes et al.-2019	TTI +120 s per vein or single 180 s in case TTI could not be measured; if TTI >90 s, applica tion aborted	2*180 s per pulmonary vein	NA	NA	14 ± 2.4	24 ± 2.8	3 and 6 months
Miyamoto et al.-2019	Single 180 s application	an additional 3-minute freeze cycle was applied to each PV after achieving PVI	146 ± 41	156 ± 44	15.5 ± 4.1	24.7 ± 4.2	12-month
Koektuerk-2019	Single 240 s application	Two 240 s applications	71.8 ± 22.8	102 ± 23.8	NA	NA	1,3,6,12 months
Vallès-2018	Double factor protocol (DFP): patients received one shot plus a bonus shot conditional to TTE and minT: TTI < 60 s + T < − 50°C: + 60 s T T I < 6 0 s + T < − 40°C: 180 s T T I < 6 0 s + T > − 40°C: 180 s + 90–180 s TTI > 60 s: 180 s + 90–180 s T < − 40°C: 180 s T > −40°C: 180 s + 180 s	patients received at least two shots of 180 s per vein.	119.8 ± 28	134.6 ± 33.7	17.5 ± 5.8	24.6 ± 6.2	Patients had follow-up at 1, 3, and 6 months after the procedure and every 6 months thereafter.
Yoshiga-2019	Single 180 s application	After a successful PVI one additional bonus freeze-cycle of a 120-s duration was applied	NA	NA	NA	NA	1,3,6,12 months
Pott-2018	TTI <30 s, application 120 s; TTI 30– 60 s, single application 180 s; TTI >60 s, 180 s application + 180 s bonus; no TTI recording, single 180 s application	Two 240 s applications	85.8 ± 27.3	115.7 ± 27.1	14.2 ± 5.1	33.5 ± 4.4	12 lead rest-ECG, and 7-day-Holter-monitoring at 1, 3, and 6 months after the procedure and thereafter every 6 months
Rottner-2018	After documentation of PVI the freeze-cycle was prolonged for additional 120 s. If no real-time PV signal recording could be obtained, a standard freeze-cycle of 180 s was applied. No bonus freeze-cycle was applied	Two 240 s applications	86.8 ± 26.1	143.5 ± 22.8	16.2 ± 6.7	24.5 ± 5.6	Not mentioned
Ströker-2018	Single 180/240 s application, give a second cryoapplication if: temperature >−40°C within 1 min or no PVI or early spontaneous PV reconnection.	1–2 bonus application(s) (240 s/180 s) after PVI	65 ± 19	106 ± 25	NA	NA	1, 3, and 6 months, and every 6 months
Mortsell-2018	Single 240 s application; either TTI or temp. <−40°C within 120 s	Two 240 s applications	99.4 ± 33.3	118.4 ± 34.3	NA	NA	at 3, 6, and 12 months
Aryana-2017	TI < 60 s,one application; TTI + 120 s; TTI 60–90 s, one application TTI + 120 s and bonus 120 s; TTI >90 s, application aborted; no TT PVI, 180 s application plus 120 s bonus	2–3 applications lasting 2–4 min	84 ± 23	145 ± 49	16 ± 5	40 ± 14	at 6-weeks, at 3, 6, 9 or 12 months
Chun-2016	TTI <75 s or within 25 s after a pull down, application 240 s; TTI >75 s or not recorded, application 240 s and 240 s bonus	Two 240 s applications	70 ± 20	89 ± 21	NA	NA	at 3, 6 and 12 months
Ekizler-2017	Single application 240 s	Two 240 s applications	67 ± 10.7	83.6 ± 10.6	NA	NA	at 1, 3, 6, 12 months, and biannually thereafter.
Tebbenjohanns-2016	a freeze cycle of 240 s was applied, adenosine challenge	Two 240 s applications	78 ± 12	93 ± 12	17.7 ± 2.9	28.8 ± 5.6	at 3, 6, 12, and 18 months
Heeger-1	Single 240 s application	1 bonus 240 s applica tion after PVI	113.8 ± 32	138.2 ± 29	NA	NA	at 3, 6, 12 months and in 6-months intervals thereafter

### 3.2. Efficacy between single-shot technique and standard technique

**Figure 2:**
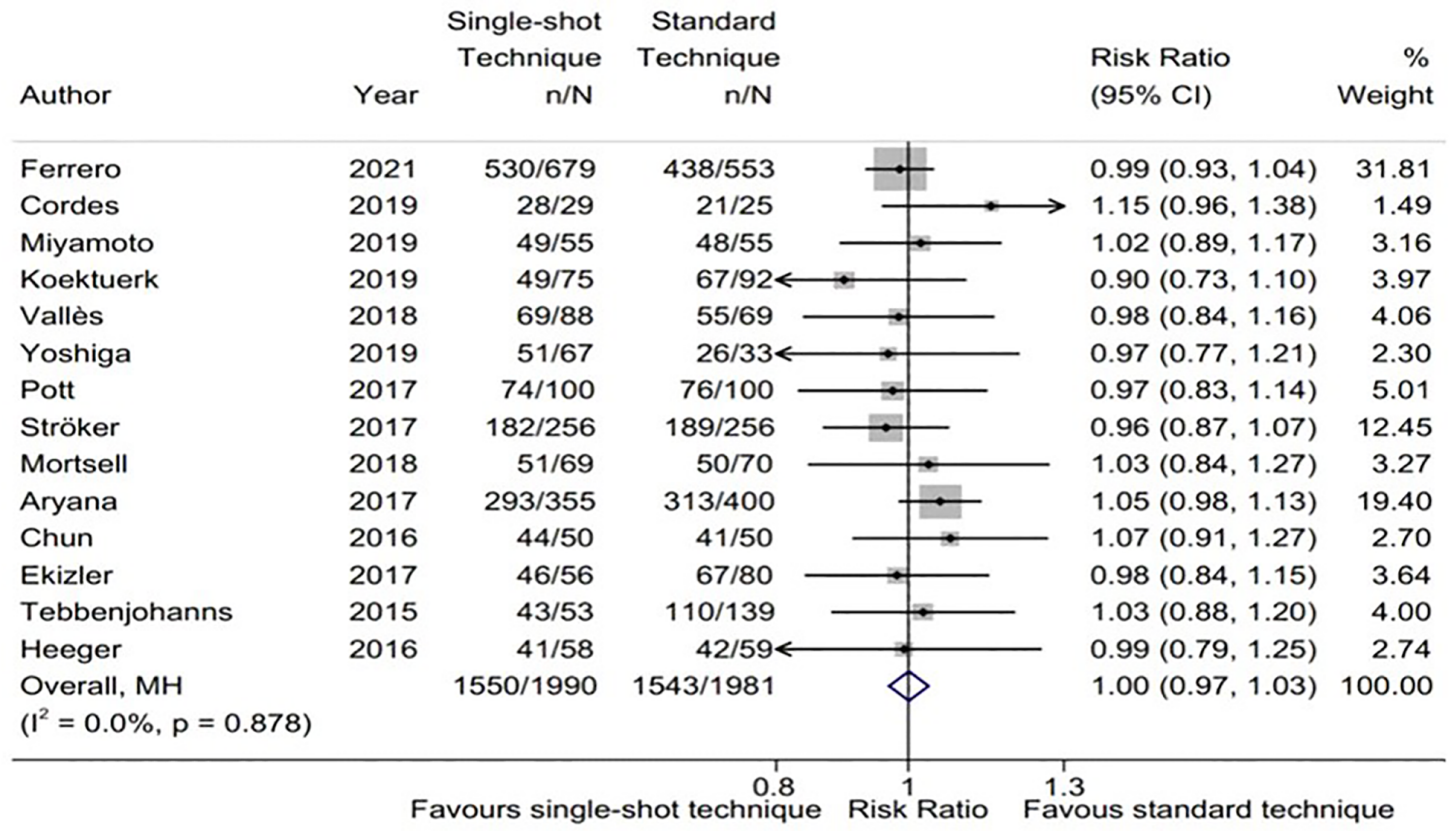
Forest plot of the freedom from atrial fibrillation (AF)/atrial tachycardia (AT). Comparison of the rate of freedom from AF/AT between single-shot technique and standard technique. AF, atrial fibrillation; AT, atrial tachycardia.

A total of 14 studies (8, 13, 14, 15, 17, 22–33) reported 3,971 patients getting rid of AF/atrial tachycardia (AT), including 1990 in the single-shot technique group and 1981 in the standard technique group. Using the random effect model, the recurrence rate of AF/AT in the single-shot technique group was similar to that in the standard technique group (RR, 1.00; 95%CI, 0.97–1.03; *P *= 0.968; *I*^2 ^= 0.0%; Figure 2).

**Figure 3:**
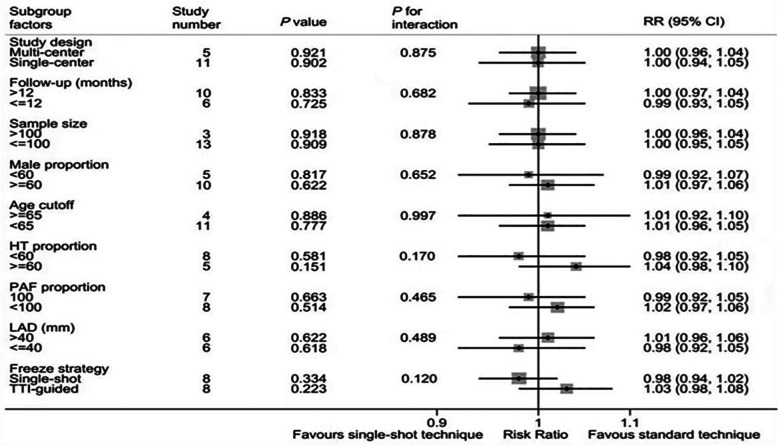
Forest plot of subgroup analysis for freedom from AF. Subgroup analysis of the rate of freedom from AF/AT between single-shot technique and standard technique.

A total of nine subgroup factors of the freedom of AF were analyzed, and the results were shown in Figure 3 and Supplementary Table S1. There was no significant statistical difference among the subgroups. Interestingly, we found that in the predetermined subgroups, the success rates of ablation in single-shot freeze and TTI-guided groups were the same, and both were comparable to those in standard technique.

Moreover, the sensitivity analysis show that there was no significant change for the pooled proportion, ranging from 0.99 (0.95–1.03) to 1.01 (0.97–1.05). Meanwhile, there was no publication bias with Egger's test (*P* = 0.94). These results suggested that our pooled results were robust.

### 3.3. Safety between single-shot technique and standard technique

**Figure 4:**
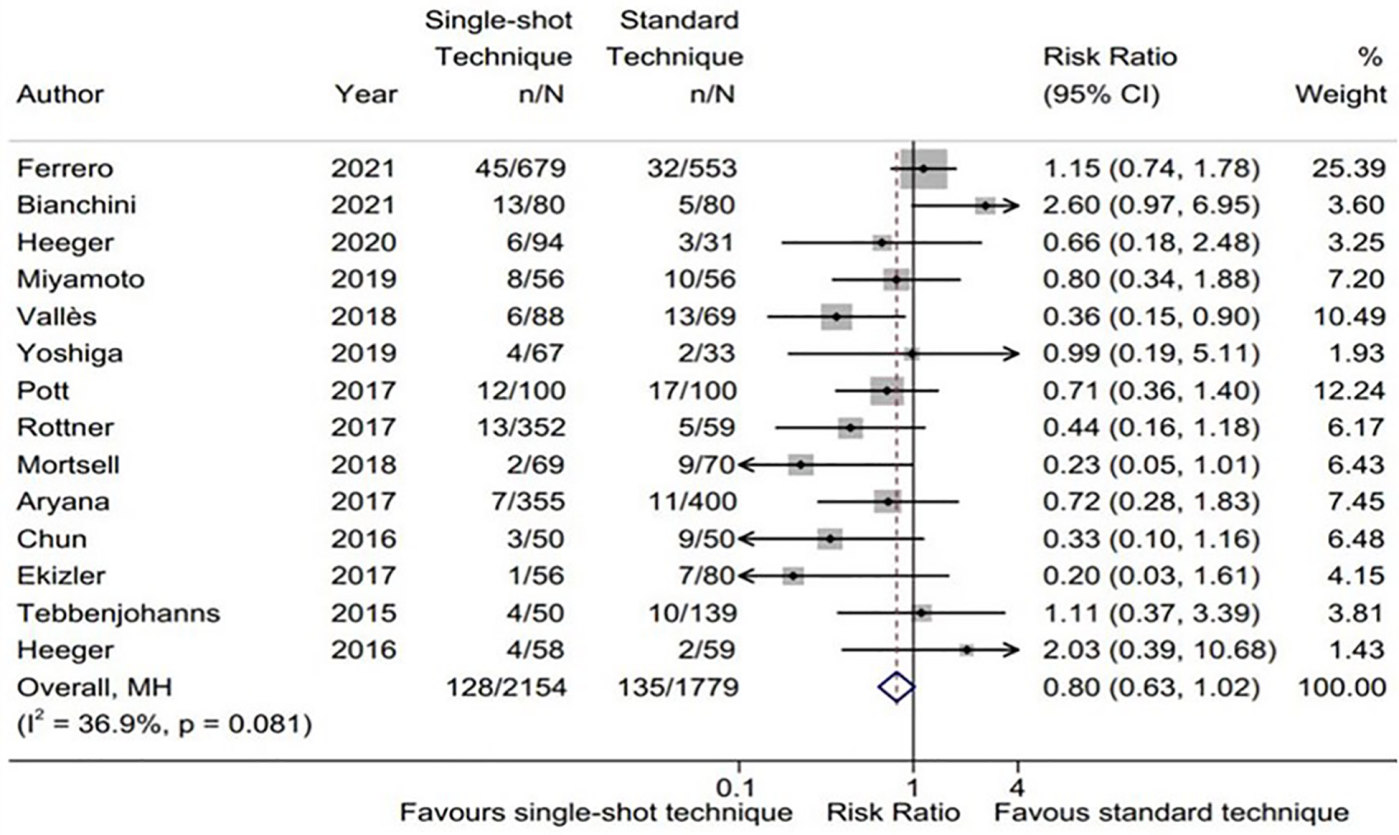
Forest plot of the procedure complications. Comparison of the procedure complications between single-shot technique and standard technique.

14 studies reported procedure complications, 13 reported transient phrenic paralysis (t-PNP), and 11 reported per-PNP. The risk of complications in single-shot technique group showed a trend for lower rate than that in standard technique group. (RR, 0.80; 95%CI, 0.63–1.02; *P* = 0.069; *I*^2 ^= 36.9%; Figure 4). Moreover, the sensitivity analysis show that there was no significant change for the pooled proportion, ranging from 0.69 (0.52–0.91) to 0.85 (0.67–1.09). Meanwhile, there was no publication bias with Egger's test (*P* = 0.18). These results suggested that our pooled results were robust.

**Figure 5:**
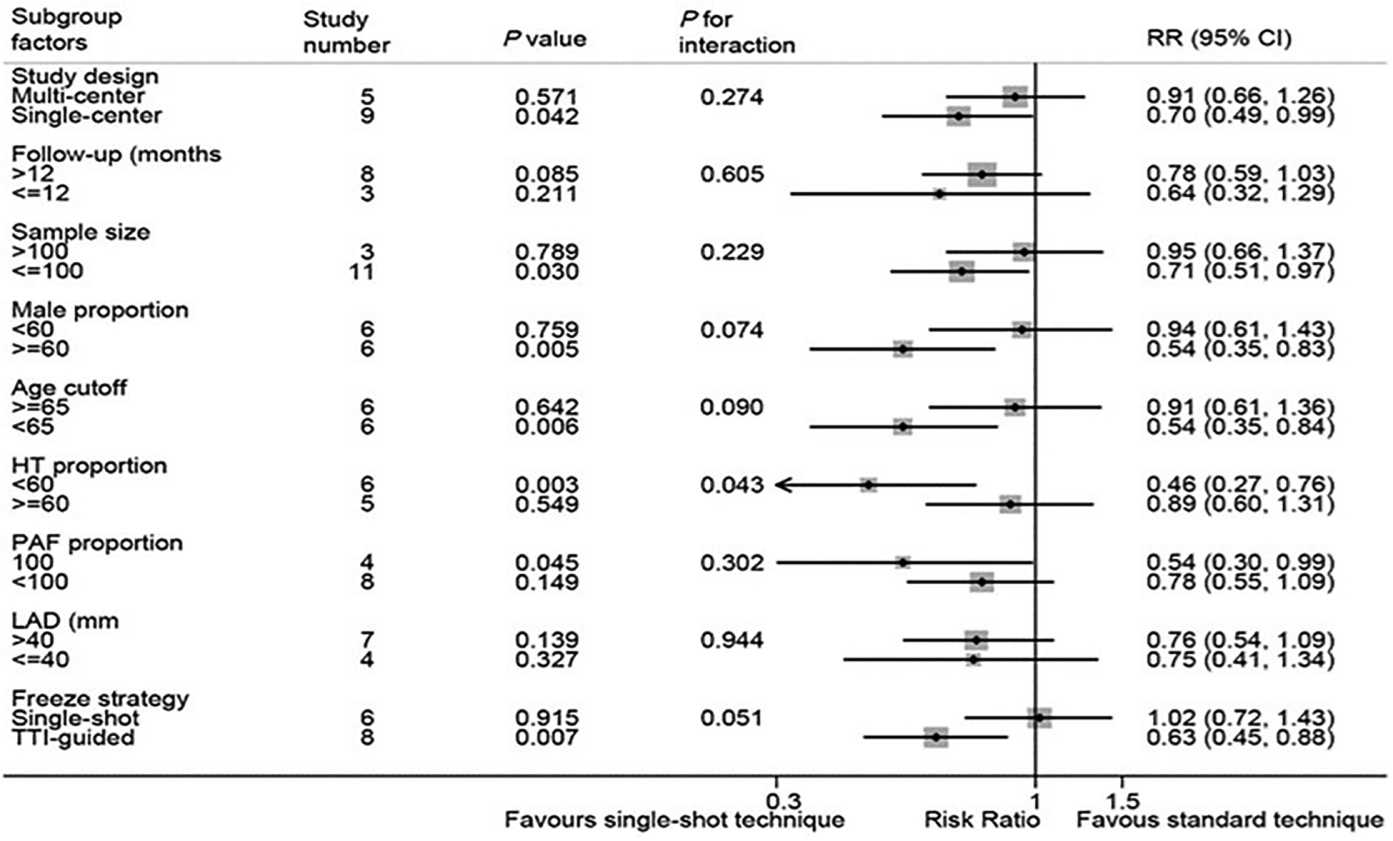
Forest plot of subgroup analysis for procedure complications. Subgroup analysis of the procedure complications between single-shot technique and standard technique.

In our meta-analysis, the subgroup analysis results are shown in Figure 5 and Supplementary Table S2. The single center subgroup (RR, 0.70; 95%CI, 0.49–0.99; *P* = 0.042; *I*^2 ^= 50.6%), sample size <100 subgroup (RR, 0.71; 95%CI, 0.52–0.97; *P *= 0.030; *I*^2 ^= 38.5%), high male proportion subgroup (RR, 0.54; 95%CI, 0.35–0.83; *P *= 0.005; *I*^2 ^= 19.9%), age <65 subgroup (RR, 0.54; 95%CI, 0.35–0.84; *P *= 0.006; *I*^2 ^= 23.3%), low proportion of HT subgroup (RR, 0.46; 95%CI, 0.27–0.76; *P *= 0.003; *I*^2 ^= 4.6%), simple paroxysmal atrial fibrillation subgroup (RR, 0.54; 95%CI, 0.30–0.99; *P *= 0.045; *I*^2 ^= 0.0%), and TTI-guided subgroup (RR, 0.63; 95%CI, 0.45–0.88; *P *= 0.007; *I*^2 ^= 45.8%) are significant statistical differences. There was no significant difference in left atrial diameter subgroup and follow-up time subgroup. Importantly, potentially significant treatment covariable interactions in the risk of complications were found in freeze strategy subgroup, male proportion subgroup and age subgroup, including single-shot freeze (RR 1.02; 95%CI, 0.72–1.43; *P *= 0.915) and TTI-guided(RR 0.63; 95%CI, 0.45–0.88; *P *= 0.007), interaction *P *= 0.051; high male proportion (RR 0.54; 95%CI, 0.35–0.83; *P *= 0.005) and a low male proportion (RR 0.94; 95%CI, 0.61–1.43; *P *= 0.759), interaction *P* = 0.074; age ≥ 65 (RR0.91; 95%CI, 0.61–1.36; *P *= 0.642) and age <65 (RR 0.54; 95%CI, 0.35–0.84; *P *= 0.006), interaction *P* = 0.090. In addition, significant treatment covariable interactions in the risk of complications were found in the hypertension subgroup, including HT > 60% (RR 0.89; 95%CI, 0.60–1.31; *P *= 0.549) and HT ≤ 60% (RR 0.46; 95%CI, 0.27–0.76; *P *< 0.01), interaction *P* = 0.043.

Compared with standard technique group, single-shot technique group had a lower incidence of t-PNP (RR, 0.67; 95%CI, 0.45–0.96; *P *= 0.038; *I*^2 ^= 0.0%). The sensitivity analysis show that there was no significant change for the pooled proportion, ranging from 0.57 (0.38–0.87) to 0.71 (0.48–1.07). No publication bias was presented with Egger's test (*P* = 0.06). These results suggested that our pooled results were robust. In addition, a similar risk of per-PNP was showed between standard technique group and single-shot technique group (RR, 1.15; 95%CI, 0.63–2.10; *P *= 0.645; *I*^2 ^= 0.0%). The sensitivity analysis, ranging from 1.00 (0.54–1.86) to 1.37 (0.71–2.65), as well as Egger's test (*P* = 0.96) also showed that our pooled results were robust.

### 3.4. Procedure parameters

Total operation time was reported in 15 studies(8, 13–17, 22–25, 27, 29–32), freeze time in 8 studies(8, 13, 15, 22, 24, 26, 29, 31)^,^ and fluoroscopy time in 14 studies(8, 13–15, 17, 22–25, 27, 30–33). The results showed that the total operation time of single-shot technique group was significantly shorter than that of standard technique group. (SMD: −25.2; 95%CI: −35.25, −15.80; *I*^2^ = 97.5%, *P *< 0.001),and the freezing application time is significantly reduced (SMD: −11.73; 95%CI: −16.33, −7.2; *I*^2^ = 99.0%, *P *< 0.001). In terms of fluoroscopy time, the single-shot technique group decreased slightly (SMD: −3.3; 95%CI: −6.03,−0.58; *I*^2^ = 96.9%, *P *< 0.001).

### 3.5. Risk of bias

Of the seventeen studies, three were randomized clinical trials (RCT) studies and the remaining fourteen were Non-RCT studies. The Quality of the eligible studies was moderate to good (Tables 3, 4). All the three RCTs lacked blinding of participants and personnel to the intervention, and all observational studies had control groups and obtained their data directly from medical records. The NOS score varied between 8 and 9 which means they were high-quality observational studies.

**Table 3:** Quality assessment for randomized clinical trials according to the cochrane risk of bias assessment tool.

First author (year)	Random sequence generation (selection bias)	Allocation concealment (selection bias)	Blinding of participants and personnel (performance bias)	Incomplete outcome data (attrition bias)	Selective reporting (reporting bias)	Other bias
Chun 2016	U	U	H	U	L	U
Miyamoto 2019	L	L	H	L	L	U
Mortsell 2018	L	L	H	L	L	U

L, Low risk of bias; H, High risk of bias; U, Uncertain.

**Table 4:** Quality assessment of enrolled studies according to the Newcastle-Ottawa quality Assessment scale (NOS).

First author (year)	Selection			Comparability		Outcome			Total stars
	Representativeness of the exposed cohort	Selection of the non-exposed cohort	Ascertainment of exposure	Demonstration that outcome of interest was not present at start of study	Comparability of cohorts on the basis of the design or analysis	Assessment of outcome	Was follow-up long enough for outcomes to occur	Adequacy of follow-up of cohorts	
Heeger 2016	★	★	★	★	★	★	★	★★	9
Tebbenjohanns 2016	★	★	★	★	★	★	★	★	8
Ekizler 2017	★	★	★	★	★	★	★	★	8
Aryana 2017	★	★	★	★	★	★	★	★	8
Ströker 2018	★	★	★	★	★	★	★	★	8
Rottner 2018	★	★	★	★	★	^a^	^a^	^a^	^a^
Pott 2018	★	★	★	★	★	★	★	★★	9
Yoshiga 2019	★	★	★	★	★	★	★	★★	9
Vallès 2018	★	★	★	★		★	★	★★	8
Koektuerk 2019	★	★	★	★	★	★	★	★★	9
Cordes 2019	★	★	★	★	★	★	★	★	8
Heeger 2020	★	★	★	★	★	^a^	^a^	^a^	^a^
Bianchini 2021	★	★	★	★	★	^a^	^a^	^a^	^a^
Ferrero 2021	★	★	★	★		★	★	★★	8

^a^ Study focused on the safety of the procedure.

### 3.6. Sensitivity analysis for RCT and Non-RCT studies

Sensitivity analysis was performed for RCT and Non-RCT studies, and the method was to recalculate the pooled OR/SMD estimates of the remaining studies after excluding each trial item by item. The results showed that there was no individual study having a significant impact on the overall estimation of any outcome expect for the sensitivity results of the procedural complications in terms of RCT (Supplementary Table S3).

## 4. Discussion

We systematically evaluated 4,688 AF patients (2,526 in single-shot technique group and 2,162 in standard technique group) derived from a total of 17 original articles. To our knowledge, this registered study had the relatively large samples to explore the ablation results of AF with different freezing strategies. The main results included (1) single-shot technique of cryoablation for AF has comparable effective and safety outcomes compared to standard technique; (2) compared with the standard technique group, the single-shot technique group showed an extent advantage in terms of the total operation time and freeze time and fluoroscopy time.

At present, there is no accepted opinion on the dose strategy for CBA. The scope of the ablation protocol has developed from a rather conservative fixed freeze cycle to an empirical bonus freeze, following with a more tailored and personalized ablation strategy to implement “time-to-isolation”. Previous studies have revealed that the single freeze showed a similar efficacy and better safety compared to the double/bonus freeze (22, 23, 27–30). Similarly, a recent meta-analysis has indicated that the single freeze strategy was as effective as the empirical double /bonus freeze strategy for CBA, while it was likely to be safer and faster (34). Unsimilar with previous studies, our registered study had the relatively large samples to explore the ablation results of AF, as well as potential determinants of AF ablation outcomes, between single-shot technique of cryoablation and standard technique. Additionally, a meta-analysis performed by Tsiachris et al. (35) showed that the personalized TTI-guided strategy based on TTI and the prolonging the duration of CBA (more than two minutes after TTI) is associated with less AF recurrence after ablation, which did not affect the safety of AF ablation and shorten the duration of ablation.

TTI, an acute biophysical indicator of PVI reflecting the transmural cold transmission, was considered as a significantly valuable and quantifiable index to evaluate the success of operation (24). Preliminary studies indicated that Cryo-application based on TTI may be significantly associated with the better long-term results (25). Recently, the INDI-FREEZE trial further reported that compared to the fixed protocol, the TTI-guided individualized approach provides a similar safety profile and clinical outcome, while reducing the total freezing time (36). It appears reasonable to implement TTI into individualized energy dosing protocols. However, the high incidence of real-time pulmonary vein potential recording is a necessary condition for the personalized PVI based on TTI. The tip length of the CB2 is 13.5 mm, which limits its real-time monitoring of TTI, and the new CB4 is characterized by a shorter tip, which may facilitate to better TTI monitoring. Although the ability of TTI monitoring had been significantly improved with the emergence of CB4, the overall capacity failed to completely satisfactory, with the success monitoring rate ranging from 70% to 86.2%. Therefore, it seemed necessary to further improve the CB system to improve the ability of TTI monitoring (37, 38). Since TTI is not acquired in each individual application, single-shot freeze still has its significance and value.

In this study, our results showed that the similar success rates of ablation were displayed in in single-shot freeze and TTI-guided group, both of which were comparable to standard technique. In relatively inexperienced centers, or even large centers with rich experience, when TTI is difficult to be recording, single-shot freeze might achieve a satisfactory success rate for AF ablation without significant increase of complications. It is worth noting that although the efficacy in single-shot freeze group and TTI-guided group was similar, the risk of complications in the TTI-guided group was significantly lower than that in the single-shot freeze group and shorten the freezing time, suggesting that personalization and optimal freeze dose are still our ultimate goals. In addition, efforts should be made to develop tools to guide ablation techniques as accurately as possible to achieve irreversible targeted myocardial tissue injury. Studies based on animal models have shown that impedance measurements of annular electrodes may provide valuable data on ice propagation. This real-time measurement may be used to guide cryopreservation applications and further reduce the risk of extracardiac injury while ensuring that PVI (39).

The confounder of gender played a significant role on CBA is still controversial. Recently, Hermida et al. (40) found that the success rate of single freeze for PVI in female patients with PAF was significantly lower than that in male patients, and the incidence of complications in female patients was higher than that in male patients. In another study, the efficacy of CBA was similar in men and women, but the risk of complications was higher in women (41). In this study, our subgroup analysis showed that there was no significant difference in the effect of sex on CBA. However, in the high male proportion subgroup, there were fewer complications in the Single-shot technique group than in standard technique group.

Our subgroup analysis showed that age had no significant effect on the efficacy of cryotherapy. Interestingly, in the age <65 subgroup, there were fewer complications in the single-shot technique group than in the standard technique group. CBA is a relatively simple procedure that can be easily applied to elderly patients. Short operation time is very important for elderly patients and can minimize the incidence of complications. Moreover, another advantage of single application over double applications is that it may reduce pain, and older patients are better tolerated. Age should not be the only factor excluding elderly patients receiving CBA for AF. As an effective and safe procedure, CBA on elderly AF patients has similar success rate and complication rate compared with young ones (42, 43). In a multi-center study for the long-term efficacy and safety of CBA in octogenarian individuals, the results showed that CBA was effective even in patients over 80 years old, with a seemingly acceptable risk profile and a lower risk of complications (44). In a large all-comer study, they found that CBA was effective in all age groups. Although more recurrence was observed in the elderly in the late stage of follow up, age was not an independent predictor of recurrence. And the incidence of perioperative adverse events is very low, and age has nothing to do with increased risk (45).

Ablation strategies with additional freeze cycles require longer freeze time, which may be associated with an increased risk of collateral damage to extracardiac structures such as phrenic nerve (PN), esophagus, and bronchial trees. In a prospective study on the endoscopic examination of mediastinal and esophageal changes caused by frozen balloon PVI, the results showed that compared with conventional protocol, TTI-guided with CB2 reduced the incidence and size of mediastinal and esophageal lesions and had a similar effect in the treatment of AF (26). Previous studies have found that less freeze application may be associated with a lower incidence of complications. Our subgroup analysis shows that single-shot technique appears to be safer for the safety of ablation, and complications of TTI-guided groups are significantly lower than those in single-shot freeze and standard technique groups. In addition, consistent with previous studies (29, 31, 32), we also found that the single-shot technique group significantly shortened the total operation time, freeze time and fluoroscopy time without increase of the procedure risk, which was beneficial to accelerate the turnover of the operating room. Interestingly, Heeger et al. (46) found that in patients with premature CB application abortion due to PNP, a high rate of persistent PVI was found at repeat procedures. It is expected to further shorten the application of freezing without affecting the success rate of ablation.

PNP is a typical complication of PVI with CBA. In the case of phrenic nerve injury, the movement of the diaphragm can be limited or even cancelled, resulting in severe dyspnea, which may counteract the clinical benefits of restoring sinus rhythm. The reported incidence of PNP is ranged from 1.1% to 6.2%. In the latest multicenter, multinational retrospective registration study, the incidence of PNP during CBA was approximately 4.2%. Overall, 97% of PNP was likely to recover within 12 months. Symptomatic and per-PNP is very rare after CBA based PVI (0.06%) (47). The recovery rate of PNP is high, and clinically related perioperative PNP seems to be rare in CBA-based PVI. Some studies had shown that there was no difference in the average numbers of freeze times between patients without PNP and patients with pers-PNP during right superior pulmonary vein isolation, suggesting that PNP had nothing to do with more cryo-application times (48). Moreover, Bianchini et al. (33) found that the number of double 120 s freeze strategy seems to be significantly less than that of a single 240 s PNP event, and only shows a short-term form of damage. However, another study showed that PNP patients had shorter freeze times and total freeze time than non-PNP patients. The authors speculate that the reason may be due to the result of the operator's emergency termination of the freeze application in the event of compound muscle action potential (CMAP) reduction or PN capture loss (49). Our meta-analysis found that compared with standard technique group, single-shot technique group had a lower incidence of t-PNP, both have a similar risk of per-PNP. We speculate that this may be due to the shorter freezing time and the more individual freezing application in the single-shot technique group. And one of the reasons for this result is the high clinical recovery rate of persistent phrenic nerve paralysis. This also indicated that the single-shot technique group reduced the incidence of phrenic nerve paralysis while shortening the operation time. Although both the left and right phrenic nerves may theoretically be injured, the right phrenic nerves are most likely to be injured during cryoablation of the right pulmonary vein because they are close to each other. Studies have shown that the use of near-end sealing technology and avoiding deeper balloon position, combined with CMAP detection is helpful to reduce the number of PNP (50).

Sensitivity analysis of effective and safety outcomes between single-shot technique vs. standard technique was performed for RCT and Non-RCT studies. The sensitivity analysis results verified the robustness of our meta-analysis results, expect the results of the procedural complications in RCT. This suggested that procedural complications between single-shot technique of cryoablation and standard technique should be interpreted with caution, and more RCTs will be needed to further confirm our results.

## 5. Limitations

Three studies focused on the safety of surgery without mentioning further follow-up, and none of the studies were achieved with five- or ten-year follow-up, making it a challenge to objectively evaluate the long-term efficacy and safety of the single-shot technique group compared with the standard technique group. The results of subgroup analysis may be limited by a limited number of available studies, which leads to the need for careful interpretation of subgroup results. Therefore, large cohort randomized controlled trials and longer follow-up are needed to further confirm the clinical results. The included study could not rule out the potential effects of different freezing duration on cryoablation (such as 240 and 180 s).

## 6. Conclusions

Our study suggested that single-shot technique of cryoablation has comparable effective and safety outcomes for AF ablation compared to standard technique.
