# Supplementary material for: Single-shot technique of cryoablation for atrial fibrillation has comparable effective and safety outcomes compared to standard technique: insights from multiple clinical studies
Source: Front Cardiovasc Med. 2023 Sep 7;10:1195492. doi: 10.3389/fcvm.2023.1195492 (PMC10512731; doi:10.3389/fcvm.2023.1195492)
Supplement: Supplementary file 1 [file Datasheet1.docx]

**Supplementary Table 1.** Subgroup analysis of freedom of AF between Single-shot Technique Versus Standard Technique

| Subgroup Factors | Numbers of Study | RR (95%CI) | I^2^ (%) | *P* value | *P* for interaction |
| --- | --- | --- | --- | --- | --- |
| Study design |  | 1.00(0.96, 1.04)  1.00(0.94, 1.05)  1.00(0.97,1.04)  0.99(0.92,1.06)  1.00(0.96,1.04)  1.00(0.94,1.05)  0.99(0.91,1.08)  1.01(0.97,1.06)  1.01(0.92,1.10)  1.01(0.96,1.06)  0.98(0.91,1.05)  1.04(0.98,1.10)  0.98(0.91,1.06)  1.02(0.97,1.06)  1.01(0.96,1.06)  0.98(0.90,1.06) |  |  | 0.842 |
| Multi-center | 4 |  | 0 | 0.402 |  |
| Single-center | 10 |  | 0 | 0.870 |  |
| Follow-up |  |  |  |  | 0.638 |
| >12 | 9 |  | 0 | 0.822 |  |
| ≤12 | 5 |  | 0 | 0.537 |  |
| Sample size |  |  |  |  | 0.905 |
| >100 | 3 |  | 30.5 | 0.918 |  |
| ≤100 | 11 |  | 0 | 0.43 |  |
| Male proportion |  |  |  |  | 0.609 |
| <60 | 4 |  | 0 | 0.961 |  |
| ≥60 | 9 |  | 0 | 0.656 |  |
| Age cutoff |  |  |  |  | 0.972 |
| ≥65 | 4 |  | 0 | 0.886 |  |
| <65 | 9 |  | 0 | 0.729 |  |
| HT proportion |  |  |  |  | 0.183 |
| <60 | 6 |  | 0 | 0.563 |  |
| ≥60 | 5 |  | 0 | 0.151 |  |
| PAF proportion |  |  |  |  | 0.470 |
| 100 | 5 |  | 0 | 0.648 |  |
| <100 | 8 |  | 0 | 0.514 |  |
| LAD(mm) |  |  |  |  | 0.454 |
| >40 | 6 |  | 0 | 0.622 |  |
| ≤40 | 5 |  | 0 | 0.565 |  |
| Freeze strategy |  |  |  |  | 0.088 |
| Single-shot | 8 | 0.98(0.94,1.02) | 0 | 0.334 |  |
| TTI-guided | 6 | 1.04(0.99,1.10) | 0 | 0.159 |  |

**Supplementary Table 2.** Subgroup analysis of procedure complications between Single-shot Technique Versus Standard Technique

| Subgroup Factors | Numbers of Study | RR (95%CI) | I^2^ (%) | *P* value | *P* for interaction |
| --- | --- | --- | --- | --- | --- |
| Study design |  | 0.91(0.66，1.26)  0.70(049, 0.99)  0.78(0.59,1.03)  0.64(0.32,1.29)  0.95(0.66,1.37)  0.71(0.51,0.97)  0.94(0.61,1.43)  0.54(0.35,0.83)  0.91(0.61,1.36)  0.54(0.35,0.84)  0.46(0.27,0.76)  0.89(0.60,1.31)  0.54(0.30,0.99)  0.78(0.55,1.09)  0.76(0.54,1.09)  0.75(0.41,1.34) |  |  | 0.274 |
| Multi-center | 5 |  | 0 | 0.571 |  |
| Single-center | 9 |  | 50.6 | 0.042 |  |
| Follow-up |  |  |  |  | 0.605 |
| >12 | 8 |  | 41.0 | 0.085 |  |
| ≤12 | 3 |  | 0 | 0.211 |  |
| Sample size |  |  |  |  | 0.229 |
| >100 | 3 |  | 41.3 | 0.789 |  |
| ≤100 | 11 |  | 38.5 | 0.030 |  |
| Male proportion |  |  |  |  | 0.074 |
| <60 | 6 |  | 31.0 | 0.759 |  |
| ≥60 | 6 |  | 19.9 | 0.005 |  |
| Age cutoff |  |  |  |  | 0.090 |
| ≥65 | 6 |  | 35.5 | 0.642 |  |
| <65 | 6 |  | 23.3 | 0.006 |  |
| HT proportion |  |  |  |  | 0.043 |
| <60 | 6 |  | 4.6 | 0.003 |  |
| ≥60 | 5 |  | 42.8 | 0.549 |  |
| PAF proportion |  |  |  |  | 0.302 |
| 100 | 4 |  | 0 | 0.045 |  |
| <100 | 8 |  | 46 | 0.149 |  |
| LAD(mm) |  |  |  |  | 0.944 |
| >40 | 7 |  | 48.6 | 0.139 |  |
| ≤40 | 4 |  | 0 | 0.327 |  |
| Freeze strategy |  |  |  |  | 0.051 |
| Single-shot | 6 | 1.02(0.72,1.43) | 0 | 0.915 |  |
| TTI-guided | 8 | 0.63(0.45,0.88) | 45.8 | 0.007 |  |

**Supplementary Table 3.** Sensitivity analysis of effective and safety outcomes between Single-shot Technique Versus Standard Technique in randomized and non-randomized studies.

| Outcomes | Type | N | Pooled RR (95%CI) | I^2^ (%) | P value | Sensitivity analysis |
| --- | --- | --- | --- | --- | --- | --- |
| Freedom of AF/AT | RCT | 3 | 1.04(0.94，1.15) | 0 | 0.427 | 1.03（0.91,1.16）-1.05（0.92,1.20） |
|  | Non-RCT | 11 | 1.00（0.96,1.03） | 0 | 0.849 | 0.99（0.96,1.03）-1.00（0.96,1.05） |
| Procedural complications | RCT | 3 | 0.47（0.25,0.87） | 26.6 | 0.016 | 0.28（0.11,0.73）-0.58（0.29,1.09） |
|  | Non-RCT | 11 | 0.89（0.69,1.15） | 35.2 | 0.366 | 0.77（0.56,1.06）-0.97（0.74,1.27） |
| Trans-phrenic nerve palsy | RCT | 3 | 0.33（0.11,1.02） | 0 | 0.053 | 0.29（0.06,1.36）-0.38（0.10,1.36） |
|  | Non-RCT | 10 | 0.74（0.49,1.13） | 0 | 0.163 | 0.64（0.40,1.00）-0.82（0.53,1.27） |
| Per-phrenic nerve palsy | RCT | 2 | 0.67（0.11,3.89） | 0 | 0.652 | 0.33（0.01,7.99）-1.00（0.15,6.85） |
|  | Non-RCT | 9 | 1.24（0.65,2.35） | 0 | 0.514 | 1.08（0.50,2.30）-1.54（0.75,3.17） |
